# Whole-Exome Sequencing: Discovering Genetic Causes of Granulomatous Mastitis

**DOI:** 10.3390/ijms26010425

**Published:** 2025-01-06

**Authors:** Beyza Ozcinar, Zeynep Ocak, Deryanaz Billur, Baris Ertugrul, Ozlem Timirci-Kahraman

**Affiliations:** 1 Department of General Surgery, Istanbul Medical Faculty, Istanbul University, 34093 Istanbul, Türkiye; drbeyza@hotmail.com; 2 Department of Medical Genetics, Medical Faculty, Istinye University, 34396 Istanbul, Türkiye; ocak.zeynep@yahoo.com; 3 Department of Molecular Medicine, Aziz Sancar Institute of Experimental Medicine, Istanbul University, 34093 Istanbul, Türkiye; deryanazbillur@gmail.com (D.B.); baris.ertugrul@istanbul.edu.tr (B.E.); 4 Department of Molecular Medicine, Institute of Graduate Studies in Health Sciences, Istanbul University, 34093 Istanbul, Türkiye

**Keywords:** granulomatous mastitis, genetic variants, whole-exome sequencing, autoimmune disease, inflammation

## Abstract

Granulomatous mastitis (GM) is a rare, benign, but chronic and recurrent inflammatory breast disease that significantly impacts physical and psychological well-being. It often presents symptoms such as pain, swelling, and discharge, leading to diagnostic confusion with malignancy. The etiology of GM remains unclear, though autoimmune and multifactorial components are suspected. This study aimed to explore the genetic underpinnings of GM using whole-exome sequencing (WES) on 22 GM patients and 52 healthy controls to identify single nucleotide variants (SNVs) and copy number variations (CNVs) potentially linked to the disease. WES analysis revealed novel SNVs in six genes: *BRCA2* (rs169547), *CFTR* (rs4727853), *NCF1* (rs10614), *PTPN22* (rs2476601), *HLA-DRB1* (seven variants), and *C3* (rs406514). Notably, most of these variants are associated with immune regulation and inflammatory pathways, supporting the hypothesis that GM is an autoimmune disease. However, all identified variants were classified as benign according to the American College of Medical Genetics and Genomics (ACMG) guidelines, necessitating further investigation into their potential functional effects. Despite conducting CNV analysis, no significant variations were identified. This study represents a foundational step in linking genetic predisposition to GM and highlights the need for integrating genetic, clinical, and functional data to better understand GM’s pathophysiology. Future research should focus on larger cohorts, functional studies, and exploring multifactorial contributors to GM, including hormonal and environmental factors.

## 1. Introduction

Granulomatous mastitis (GM) is a rare, chronic inflammatory breast disease characterized by granuloma and abscess formation. Clinically and radiologically, it can be misdiagnosed as breast cancer, leading to unnecessary anxiety and invasive procedures [1,2]. GM predominantly affects women in their 30s and 40s, particularly those with a history of childbirth and breastfeeding. The disease significantly impacts patients’ quality of life by causing persistent pain, swelling, and psychological distress [3,4].

The etiology of GM remains unclear, with multiple factors proposed [5,6,7,8,9,10]. Autoimmune mechanisms are strongly implicated, as GM often coexists with other autoimmune diseases, such as systemic lupus erythematosus (SLE) and Sjögren’s syndrome. This phenomenon, known as polyautoimmunity, suggests shared immunological pathways among these conditions. In addition, hormonal, environmental, and infectious factors have been hypothesized, indicating a multifactorial nature. For example, retained ductal secretions causing epithelial damage and subsequent immune activation are considered possible triggers. However, the lack of comprehensive studies has hindered definitive conclusions about GM’s pathophysiology [11,12,13,14,15].

Genetic predisposition may play a critical role in GM, particularly through variants in immune-related genes. Previous studies have highlighted the involvement of specific *HLA* alleles in autoimmune conditions, but their relevance to GM remains underexplored. This study aims to address this gap by identifying single nucleotide variants (SNVs) and copy number variations (CNVs) in GM patients using whole exome sequencing (WES). By analyzing genetic data and their potential clinical significance, we seek to enhance understanding of GM’s molecular mechanisms and contribute to the development of targeted therapies.

## 2. Results

### 2.1. Population Characteristics

This study explored the genetic basis of GM by analyzing a cohort of 22 patients diagnosed with GM and 52 healthy controls. Patients were evaluated at Istanbul University Medical Faculty, with detailed clinical, imaging, and biopsy data. Controls were selected based on matching criteria, including age, sex, and ethnicity, to minimize confounding genetic variability. Table 1 summarizes the clinical characteristics of the GM cohort. Imaging findings have been standardized using Breast Imaging Reporting and Data System nomenclature to improve clarity and consistency.

### 2.2. Next Generation Sequencing Analysis

WES identified 12 SNVs across six genes: *BRCA2*, *CFTR*, *NCF1*, *PTPN22*, *HLA-DRB1*, and *C3*. Seven variants were found in the *HLA-DRB1* gene, while the remaining five were distributed among other genes. Table 2 summarizes the detected SNVs, their minor allele frequencies (MAFs), and associated statistical measures.

SNVs were identified in 18/22 GM patients (81.8%) and 10/52 controls (19.2%). Detailed data are provided in Table 2. For example, rs10614 (*NCF1*) showed an odds ratio (OR) of 4.2 (*p* = 0.002), suggesting a potential association with GM. However, several variants, such as rs169547 (*BRCA2*) and rs9270303 (*HLA-DRB1*), have high MAFs (MAF > 0.5) in the general population.

Deeply looking into our results, we identified the following variants: rs10614 c.295 A > C/A > G/A > T (p.Ser99=) in homozygous patients in the *NCF1* gene, rs4727853 c.1251C > A (p.Asn417Lys) in heterozygous patients in the *CFTR* gene, rs169547 c.7397T > C (p.Val2466Ala) in homozygous patients in the *BRCA2* gene, rs2476601 c.1858= (p.Trp620=) in homozygous patients in the *PTPN22* gene, and rs406514 c.155-8C > T in homozygous patients in the *C3* gene.

Out of the twelve variants, seven were in *HLA-DRB1*. These were rs9270303, rs2308760, rs3830130, rs707953, rs701829, rs9269958, and rs9270299. Population frequencies varied, with some SNVs being quite common (e.g., rs10614 in *NCF1* at 70.9%) and others being less frequent (e.g., rs4727853 in *CFTR* at 30.7%). Eight SNVs were identified with a MAF greater than 0.5, signifying that the minor allele is present in more than half of the population for these SNVs. These SNVs are rs169547 (*BRCA2*), rs10614 (*NCF1*), rs2476601 (*PTPN22*), rs9270303 (*HLA-DRB1*), rs3830130 (*HLA-DRB1*), rs9270299 (*HLA-DRB1*), rs701829 (*HLA-DRB1*), and rs406514 (*C3*). On the other hand, four SNVs, rs4727853 (*CFTR*), rs2308760 (*HLA-DRB1*), rs707953 (*HLA-DRB1*), and rs9269958 (*HLA-DRB1*), stand out with population frequencies below 0.5 (0.307, 0.384, 0.481, and 0.467, respectively). All twelve SNVs were classified as benign according to the American College of Medical Genetics and Genomics (ACMG) classification [16]. ClinVar information, which provides additional details on variant pathogenicity, was not available for all SNVs. In ClinVar, the *CFTR* gene is related to cystic fibrosis (CF), the *BRCA2* gene is associated with breast cancer and breast-ovarian cancer syndrome, and the *C3* gene is associated with complement component 3 deficiency. In Online Mendelian Inheritance in Man (OMIM), the *NCF1* gene is associated with chronic granulomatous disease, the *CFTR* gene is associated with cystic fibrosis and hereditary pancreatitis, the *BRCA2* gene is associated with male breast cancer, medulloblastoma, prostate cancer, and pancreatic cancer, the *PTPN22* gene is associated with SLE, diabetes mellitus type 1, and rheumatoid arthritis susceptibility, the *C3* gene is associated with age-related macular degeneration and *C3* deficiency, and the *HLA-DRB1* gene is associated with multiple sclerosis and sarcoidosis susceptibility (Table 2).

CNV analysis was performed using ExomeDepth v1.1.16 software. No significant variations were detected. The results suggest that larger datasets may be required to detect subtle CNV effects.

### 2.3. Sanger Sequencing Verification

To validate the WES findings, Sanger sequencing (SS) was performed on a subset of variants identified in the *BRCA2*, *CFTR*, *NCF1*, *PTPN22*, and *C3* genes. The variants c.7397T > C (p.V2466A) in *BRCA2*, c.1251C > A (p.N417K) in *CFTR*, and c.295G > A (p.G99S) in *NCF1* were successfully validated by SS. SS traces confirmed that the nucleotide changes were consistent with the WES results, supporting the reliability of these findings. Variants in *HLA-DRB1* were not subjected to SS due to resource limitations, highlighting the need for further validation in future studies.

## 3. Discussion

GM is an uncommon, long-lasting inflammatory breast disease with an unclear cause. While the exact trigger remains a mystery, some evidence suggests it might be related to the body’s immune system attacking healthy breast tissue [8,17]. This study aimed to explore the genetic basis of GM through WES and identified novel SNVs in six key genes: *BRCA2*, *CFTR*, *NCF1*, *PTPN22*, *HLA-DRB1*, and *C3*. These findings provide preliminary evidence supporting the hypothesis that GM may be influenced by genetic predisposition, particularly through immune-related pathways. Both our study and that of Zhu et al. [17] provide valuable insights into the pathophysiology of GM, albeit through different approaches. Our study focused on identifying genetic variations through whole-exome sequencing, revealing novel SNVs in genes primarily involved in immune regulation. This aligns with Zhu et al.’s findings, which demonstrated significant enrichment of differentially expressed mRNAs in immune-related pathways. While our study primarily explored genetic predispositions, Zhu et al. delved into the transcriptional landscape of GM, identifying potential drug targets such as *HSD11B1*, a gene linked to the efficacy of prednisone, a common GM treatment. Both studies underscore the crucial role of the immune system in GM pathogenesis and highlight the need for further research to elucidate the complex interplay of genetic, transcriptional, and environmental factors contributing to this challenging condition.

Considering all of these research data, it appears that our hypothesis that GM is an autoimmune disease is consistent with the findings. This is supported by the effective steroid treatment of GM, as stated by Zhu et al. [17], and the fact that GM is a disease caused by dysfunctions of the immune system.

Additionally, there are some studies showing the simultaneous appearance of GM with other autoimmune diseases such as SLE and Sjögren’s syndrome [12,13,14]. This simultaneous occurrence of two or more autoimmune diseases in a patient is called polyautoimmunity and is considered a common phenomenon in rheumatic and autoimmune diseases [18,19,20]. The presence of polyautoimmunity in GM patients highlights the importance of shared etiopathogenesis with other autoimmune diseases.

Environmental and hormonal factors are suspected to play a role; the potential contribution of genetics remains unclear [21]. This study aimed to shed light on this by analyzing single nucleotide variants and CNVs in GM patients compared to healthy controls. We identified specific variants in genes linked to autoimmune and inflammatory processes— *BRCA2* (rs169547), *CFTR* (rs4727853), *NCF1* (rs10614), *PTPN22* (rs2476601), *HLA-DRB1* (rs9270303, rs2308760, rs3830130, rs707953, rs701829, rs9269958, rs9270299), and *C3* (rs406514). The finding of SNVs in *NCF1* [22], *CFTR* [23], and *PTPN22* [24] is particularly interesting due to the established roles in immune function and inflammatory processes. The detection of these SNVs in GM patients suggests a potential link between genetic predisposition to altered immune function and the inflammatory processes characteristic of GM. This study provides preliminary evidence linking these SNVs to GM susceptibility in humans.

We report a novel association of the *NCF1* gene variant rs10614 with GM. *NCF1* plays a critical role in immune function through the production of reactive oxygen species (ROS) by white blood cells [25,26]. Reduced ROS production due to *NCF1* variants is linked to autoimmunity in diseases like SLE [22,27]. While the precise mechanism by which rs10614 contributes to GM requires further investigation, the observed association suggests a potential link between this variant, altered ROS production, and the development of GM. *NCF1* is a complex gene region with pseudogenes and variable copy numbers influencing autoimmune risk across ethnicities [28,29]. Further research is needed to understand the functional consequences of rs10614 and how *NCF1* variants interact with other genes in GM development [30].

Limited research exists on *CFTR* variants and GM. We found a novel SNV (rs4727853) in the *CFTR* gene of GM patients. While the precise role of rs4727853 remains unclear, the established link between *CFTR* dysfunction and inflammation in cystic fibrosis [31] and autoimmune pancreatitis [32] suggests a potential connection to GM, a disease with possible autoimmune features supported by the presence of T-cells [33,34]. *CFTR* deficiency in macrophages is known to contribute to an imbalanced Th2-skewed immune response [35], potentially relevant to GM’s inflammatory pathology. Studies of T-cell involvement in GM further support this exploration [33,34]. Further research is crucial to understand how *CFTR* variants, including rs4727853, might influence immune response and contribute to GM development, especially in patients with steroid-resistant disease [36]. While the hypothesis that rs4727853 and GM are linked is grounded in the known involvement of *CFTR* in other diseases, this article fails to provide concrete evidence directly connecting *CFTR* deficiency to GM. To definitively establish whether this variant influences immunological signaling or *CFTR* protein function in GM patients and to confirm its relevance to the disease, conducting functional studies is essential.

The *PTPN22* gene plays a crucial role in regulating immune responses, and variations within this gene have been implicated in the development of various autoimmune diseases [37]. This study investigates a potential association between the rs2476601 (Arg620Trp) variant in *PTPN22* and GM. While previous research has linked this variant to other autoimmune disorders, it is important to note that these findings do not necessarily imply the same underlying mechanism in GM. While the rs2476601 variant may disrupt signaling pathways, potentially leading to an inflammatory response as observed in other autoimmune contexts [38], its specific impact on GM pathogenesis requires further investigation. Notably, this variant is predicted to reduce the activity of LYP, a protein crucial for the development and function of regulatory T cells (Tregs), which play a critical role in maintaining immune tolerance [39]. Given the evidence of T cell dysregulation in GM patients [33,34,40,41], we hypothesize that the rs2476601 variant may contribute to the pathogenesis of GM by impairing Treg function through reduced LYP activity. However, it is crucial to acknowledge that the precise mechanisms by which this variant might contribute to GM pathogenesis remain to be elucidated. Our findings provide preliminary evidence for an association between rs2476601 and GM, and further investigations, including functional studies, are necessary to confirm the impact of rs2476601 on Treg cell development and function in the context of GM. Investigating this mechanism could improve our understanding of GM and potentially lead to new treatments.

Our analysis revealed a previously unreported link between a *BRCA2* variant, rs169547, and GM susceptibility. *BRCA2*, a known tumor suppressor gene for breast and ovarian cancers, also plays a role in immune response [42]. The variant (rs169547) may influence immune function and GM risk, although its high frequency suggest it may not be a major disease-causing factor alone [43]. Interestingly, clinical data suggests a link between GM and breast cancer risk [44]. While further research is needed to confirm this association, rs169547 could potentially serve as a marker for increased breast cancer risk in GM patients.

This study uncovered seven novel *HLA-DRB1* gene variants associated with GM. The HLA system plays a critical role in immune response, and variations can influence autoimmune diseases [45]. Our findings add to limited research on *HLA* variants in GM. Previous studies reported associations between specific HLA markers and GM [46] and upregulated *HLA-DRB1* proteins in GM tissue [8]. Our study emphasizes the genetic underpinnings of GM by identifying novel *HLA-DRB1* variants potentially associated with the disease’s etiology. While we found a vast number of *HLA-DRB1* variations (over 8000 SNVs and indels), limited information exists in ClinVar, a key database for variant interpretation. This highlights the need for functional studies to understand how these variants might influence GM susceptibility or progression.

A novel *HLA-DRB1* variant (rs9270303) was identified in this study as a potential contributor to GM. Interestingly, this variant has been linked to Alzheimer’s disease [47] and Hepatitis B Virus (HBV)-associated hepatocellular carcinoma (HCC) [48], suggesting a potential broader role beyond GM. Additionally, research suggests population-specific prevalence of rs9270303, with Lithuanians having a higher frequency [49].

This study links rs2308760, a nonsynonymous variant in *HLA-DRB1*, to GM. While *HLA-DRB1* variants have been extensively studied in various autoimmune disorders, this represents one of the first reports associating rs2308760 specifically with GM. This finding contributes to a growing body of evidence supporting the role of *HLA-DRB1* in the pathogenesis of GM. While classified as benign, the lack of prior links to GM necessitates further investigation. Functional studies are crucial to understand how this variant might influence protein function and contribute to GM susceptibility.

While our study links the intronic *HLA-DRB1* variant rs3830130 to GM, its benign classification and lack of prior associations necessitate functional studies. These studies should investigate potential effects on splicing, regulatory elements, or gene expression in GM-relevant cell types.

Results from this study suggest that the *HLA-DRB1* variant (rs707953) may be a potential risk factor for GM. The variant shows population-specific frequencies, with Lithuanians having a higher prevalence [49]. This amino acid change necessitates functional studies to understand its impact on protein function and potential contribution to GM susceptibility. Additionally, investigating variant prevalence in GM across diverse populations could reveal links between genetic background, variant frequency, and disease risk, similar to regional variations observed in GM itself [21,34,50,51,52].

Our analysis identified a novel association between a common nonsynonymous variant (rs701829) in *HLA-DRB1* and GM. While the variant is prevalent, its impact on GM is unclear.

A stop-gain variant (rs9269958) within *HLA-DRB1* was identified by our study as a novel risk factor for GM. This moderately common variant has also been linked to asthma [53], suggesting a potential shared immune dysfunction pathway. The stop-gain variant likely disrupts antigen presentation, potentially contributing to GM and asthma. However, prior studies in rheumatoid arthritis [54] suggest the variant might be linked to a broader autoimmune susceptibility.

The presence of the rs9270299 genetic variant in GM raises questions about its potential role in the disease. Notably, research by Yu et al. and Brant et al. links *HLA* genes, particularly *HLA-DRB1*, to inflammatory bowel disease and HBV-associated HCC, respectively [48,55]. These findings suggest a broader role for *HLA*-related genes in various inflammatory conditions, potentially including GM.

*C3* variant rs406514 association with GM suggests a potential role for *C3* in disease pathogenesis. Deangelis et al. linked this variant to neovascular age-related macular degeneration, suggesting potential roles for shared inflammatory pathways influenced by *C3* variations [56]. Notably, rs406514 resides near exon 18, a functionally relevant region identified by Lokki et al. warranting further investigation into its functional impact on *C3* and its contribution to GM [57].

A substantial proportion of GM patients exhibited SNVs compared to controls. Notably, the rs10614 variant within the *NCF1* gene demonstrated a strong association with GM, as indicated by a significant odds ratio. However, several other identified variants, including those within the *BRCA2* and *HLA-DRB1* genes, displayed high frequencies within the general population. These high frequencies raise concerns regarding their specific involvement in GM pathogenesis. Therefore, further statistical analysis and functional studies are essential to definitively establish the role of these variants in the development of GM.

While all identified variants were classified as benign according to ACMG guidelines, their potential functional roles in GM pathogenesis cannot be entirely disregarded. For instance, the *NCF1* variant (rs10614) has been associated with reduced ROS production [22,27], which could impair neutrophil function and contribute to chronic inflammation observed in GM. However, experimental evidence is necessary to confirm the specific impact of this variant on GM pathology. Similarly, dysregulation of *CFTR*, as observed with the rs4727853 variant, has been linked to altered macrophage activity and immune responses in conditions like cystic fibrosis [31], suggesting a potential role in GM, particularly in steroid-resistant cases. Furthermore, the *PTPN22* variant (rs2476601) is known to affect Treg function [39], potentially disrupting immune homeostasis, a mechanism that could contribute to the inflammatory environment observed in GM. These observations highlight the need for further functional studies to elucidate the potential impact of these benign variants on GM pathogenesis and to better understand their contribution to the disease.

## 4. Materials and Methods

### 4.1. Patients and Study Approval

This study included 22 female patients diagnosed with idiopathic GM and 52 age- (37.18 ± 7.15 years), sex-, ethnicity-, and breastfeeding history-matched healthy female controls. Patients were recruited from the outpatient clinic of the Department of General Surgery at Istanbul University Medical Faculty. Detailed clinical histories, including any history of rheumatological disease and medication use, were obtained from all participants. Diagnosis of GM was confirmed through imaging (ultrasound, mammography, or MRI), histopathological examination, and exclusion of tuberculosis via chest imaging, PPD testing, and tuberculous quantiferon assays. This study adhered to the Declaration of Helsinki guidelines and was approved by the Institutional Ethics Committee of Istanbul University Faculty of Medicine (approval number: 2018/1515). Informed consent was obtained from all participants prior to sample collection. The project was supported by the Scientific Research Project Coordination Unit of Istanbul University (grant number: 38474).

### 4.2. Sample Collection

Blood samples were collected from 22 GM patients and 52 controls using Ethylenediaminetetraacetic acid, also called EDTA, tubes and stored at −20 °C. Tissue samples were exclusively used for histopathological examination. Genomic DNA was extracted from blood samples using QIAamp genomic DNA extraction kits (QIAGEN, Hilden, Germany) following the manufacturer’s protocol. DNA quality and quantity were assessed using a NanoDrop™ Lite Spectrophotometer (Thermo Fisher Scientific, Waltham, MA, USA) and visualized on a 1% agarose gel with CYBR Safe dye (Thermo Fisher Scientific, Waltham, MA, USA). Extracted DNA was stored at −80 °C until further analysis.

### 4.3. Whole-Exome Sequencing (WES)

WES was performed on 22 GM patients and 52 controls using Agilent’s SureSelect Human All Exon V6 Kit (Agilent Technologies, Inc., Santa Clara, CA, USA) for library preparation [58]. Sequencing was conducted on an Illumina NovaSeq6000 platform (Illumina, Inc., San Diego, CA, USA) with 150 bp paired-end reads, achieving an average coverage depth of 100×. Quality control measures included read depth analysis (>97% bases covered at 10×) using FastQC v0.12.1 software. Low-quality reads and adapter sequences were removed prior to variant calling.

Variants were annotated and analyzed using databases and in silico tools, including gnomAD [59], ClinVar [60], dbSNP [61], OMIM [62], PolyPhen2 [63], and SIFT [64]. The Human Genome Variation Society nomenclature was applied via the VarSeq transcript annotation algorithm. CNVs were analyzed using ExomeDepth v1.1.16 software.

### 4.4. Sanger Sequencing (SS)

SS was used to validate selected variants identified through WES in the *BRCA2*, *CFTR*, *NCF1*, *PTPN22*, and *C3* genes. Primers were designed using Primer3Plus v3.3.0 software (Table 3). Polymerase chain reaction products were purified and sequenced on an ABI genetic analyzer using the BigDye Terminator v3.1 Cycle Sequencing kit (Thermo Fisher Scientific, Waltham, MA, USA). Sequence data were analyzed to confirm the nucleotide changes detected by WES, with electropherograms visually inspected for consistency.

### 4.5. Statistical Analysis of Genetic Variants

The statistical significance of identified SNVs was evaluated using chi-square tests and logistic regression analysis. *p*-values less than 0.05 were considered statistically significant. ORs and 95% confidence intervals were calculated for each variant to assess their association with GM. CNV analysis was performed using ExomeDepth v1.1.16 software, with a false discovery rate threshold of 5%.

## 5. Conclusions

This study identified novel SNVs in genes associated with immune regulation and inflammation, including *BRCA2*, *CFTR*, *NCF1*, *PTPN22*, *HLA-DRB1*, and *C3*, in patients with GM. While all identified variants were classified as benign according to ACMG guidelines, these findings suggest a potential genetic predisposition to GM, supporting the hypothesis that GM is an autoimmune disease. However, the association of GM with benign genetic variants highlights the multifactorial nature of the disease. Hormonal, environmental, and infectious factors likely interact with these genetic predispositions, emphasizing the need for further research, integrating genetic, clinical, and environmental data to gain a more comprehensive understanding of GM.

Functional studies are essential to determine how these genetic variations impact protein function and immune pathways. Furthermore, larger and more diverse patient groups are needed to validate these findings and potentially identify additional genetic contributors. The current approach to treating GM, often involving short-term high-dose steroids, may not address the underlying autoimmune nature of the disease and can lead to significant side effects. A longer-term treatment strategy with lower-dose immunosuppressive medications, similar to that used for other autoimmune diseases, may be more appropriate. However, it is important to note that this is a hypothetical approach, and further clinical research and functional evidence are necessary to support its use in GM.

Ultimately, these research efforts could pave the way for personalized treatment strategies that target specific genetic and immunological pathways involved in GM.

### Limitations and Future Directions

Despite laying the groundwork for understanding the genetic basis of GM, this study has several limitations that should be acknowledged. The small sample size (22 patients, 52 controls) limits the statistical power of the findings and restricts their generalizability to broader populations. Larger cohorts with more diverse demographics are necessary to confirm the observed associations and explore potential population-specific genetic contributions.

Another limitation is the absence of functional studies to evaluate the impact of the identified SNVs on protein function and immune pathways. While in silico tools provide valuable insights, experimental validation (e.g., gene expression studies, protein function assays) is essential to establish causative links between these variants and GM.

Additionally, while CNV analysis was performed, no significant findings were reported. Further studies with larger datasets may help uncover subtle CNV effects that contribute to GM pathogenesis.

Future research should prioritize the following:

1. Expanding the study population to include diverse ethnicities and larger sample sizes.
2. Conducting functional studies to explore the biological effects of the identified variants.
3. Investigating the interaction between genetic, hormonal, environmental, and infectious factors in GM development.
4. Integrating genetic data with clinical phenotypes to develop personalized treatment strategies.
5. Exploring the therapeutic potential of low-dose immunosuppressive drugs in well-designed clinical trials.

By addressing these limitations, future studies can provide a more comprehensive understanding of GM’s genetic and molecular basis, paving the way for improved diagnostic tools and targeted therapies.

## Figures and Tables

**Table 1:** Clinical characteristics of the patients.

	Clinical Features	Erythema Nodosum	Ultrasound Features	Drug-Taking History	Recurrence
**1**	Bilateral multiple abscesses with sinus tracts	Yes	Multiple breast abscesses	Methylprednisolone per oral + intralesional	Yes—repeated drug therapy than surgical excision
**2**	Unilateral, large, inflamed, hard, painful breast mass	No	Diffuse hypoechoic heterogenous lesion with multiple abscesses	Methylprednisolone per oral	Yes—intralesional ozone therapy
**3**	Unilateral big mass with abscess	No	Huge abscess with inflammation around	Abscess drainage + Methylprednisolone per oral	No
**4**	Unilateral, large, inflamed, hard, painful breast mass	No	Leafy-type irregular hypoechoic lesion	Methylprednisolone per oral + intralesional	No
**5**	Bilateral, multiple, inflamed, hard, painful breast lesions with sinus tracts	No	Leafy-type multiple irregular hypoechoic lesions with small abscesses	Methylprednisolone per oral + intralesional	No
**6**	Unilateral, hard, painless breast mass	No	Localized hypoechoic mass	Observation	No
**7**	Unilateral, large, inflamed, hard, painful breast mass with sinus tract	No	Leafy-type irregular hypoechoic lesion with small abscesses	Methylprednisolone per oral	Yes—repeated drug therapy with per oral methylprednisolone
**8**	Unilateral, large, inflamed, hard, painful breast mass	No	Leafy-type irregular hypoechoic lesion	Methylprednisolone per oral	No
**9**	Bilateral, multiple, inflamed, hard, painful breast lesions	No	Leafy-type multiple irregular hypoechoic lesions	Methylprednisolone per oral	Yes—Colchicine treatment
**10**	Bilateral, multiple, inflamed, hard, painful breast lesions with sinus tracts	No	Leafy-type multiple irregular hypoechoic lesions with small abscesses	Methylprednisolone per oral	Yes—repeated drug therapy with per oral methylprednisolone
**11**	Unilateral, large, inflamed, hard, painful breast mass	No	Leafy-type irregular hypoechoic lesion	Methylprednisolone per oral	No
**12**	Unilateral, inflamed, painful breast mass	No	Heterogenous irregular hypoechoic lesion	Observation	No
**13**	Unilateral, large, inflamed, hard, painful breast mass	No	Leafy-type irregular hypoechoic lesion	Methylprednisolone per oral	No
**14**	Unilateral, large, hard breast mass	No	Large hypoechoic heterogenous irregular lesion	Methylprednisolone per oral	Yes—repeated drug therapy with per oral methylprednisolone
**15**	Unilateral, large, inflamed, hard, painful breast mass	No	Leafy-type irregular hypoechoic lesion	Methylprednisolone per oral	Yes—repeated drug therapy with per oral methylprednisolone
**16**	Unilateral, hard breast mass with no inflammation	No	Hypoechoic irregular mass lesion	Surgical excision	No
**17**	Unilateral, inflamed, painful breast mass	No	Heterogenous irregular hypoechoic lesion	Methylprednisolone per oral	No
**18**	Unilateral, large, inflamed, hard, painful breast mass with abscess	No	Leafy-type irregular hypoechoic lesion with abscess	Abscess drainage + Methylprednisolone intralesional	Yes—methotrexate ebewe per oral
**19**	Bilateral, multiple, inflamed, hard, painful breast lesions with sinus tracts	No	Leafy-type multiple irregular hypoechoic lesions with small abscesses	Triamcinolone acetonide intralesionally + externally	Yes—repeated drug therapy with per oral methylprednisolone + triamsinolon asetonid intralesional than methotrexate ebewe per oral
**20**	Unilateral, multiple, inflamed, hard, painful breast lesions with sinus tracts	No	Leafy-type multiple irregular hypoechoic lesions with small abscesses	Triamcinolone acetonide intralesionally + externally	Yes—repeated drug therapy with per oral methylprednisolone + triamsinolon asetonid intralesional than methotrexate ebewe per oral
**21**	Unilateral, large, inflamed, hard, painful breast mass	No	Leafy-type irregular hypoechoic lesion	Triamcinolone acetonide intralesionally + externally	Lost in follow-up
**22**	Unilateral, inflamed, painful breast mass	No	Heterogenous irregular hypoechoic lesion	Methylprednisolone intralesional	No

**Table 2:** Candidate genetic variants associated with granulomatous mastitis.

Gene Symbol	Chromosome	Genotype	dbSNP	Population Frequency	Translation Impact	ACMG	ClinVar	OMIM
*BRCA2*	13	Hom (141/141)	rs169547	0.982	nonsynonymous SNV	Benign	breast cancer	Male breast cancer, medulloblastoma, prostate cancer, pancreatic cancer
*CFTR*	7	Het (40/93)	rs4727853	0.307	nonsynonymous SNV	Benign	cystic fibrosis	Cystic fibrosis, hereditary pancreatitis
*NCF1*	7	Hom (121/130)	rs10614	0.709	nonsynonymous SNV	Benign	not specified	Chronic granulomatous disease
*PTPN22*	1	Hom (59/59)	rs2476601	0.918	nonsynonymous SNV	Benign	no information	Systemic lupus erythematosus, diabetes mellitus type 1, and rheumatoid arthritis susceptibility
*HLA-DRB1*	6	Hom (322/322)	rs9270303	0.76	nonsynonymous SNV	Benign	no information	Multiple sclerosis and sarcoidosis susceptibility
*HLA-DRB1*	6	Het (139/316)	rs2308760	0.384	nonsynonymous SNV	Benign	no information	Multiple sclerosis and sarcoidosis susceptibility
*HLA-DRB1*	6	Het (159/173)	rs3830130	0.709	intron	Benign	no information	Multiple sclerosis and sarcoidosis susceptibility
*HLA-DRB1*	6	Het (134/299)	rs707953	0.481	nonsynonymous SNV	Benign	no information	Multiple sclerosis and sarcoidosis susceptibility
*HLA-DRB1*	6	Hom (317/317)	rs701829	0.839	nonsynonymous SNV	Benign	no information	Multiple sclerosis and sarcoidosis susceptibility
*HLA-DRB1*	6	Het (35/85)	rs9269958	0.467	stopgain	Benign	no information	Multiple sclerosis and sarcoidosis susceptibility
*HLA-DRB1*	6	Hom (214/214)	rs9270299	0.839	nonsynonymous SNV	Benign	no information	Multiple sclerosis and sarcoidosis susceptibility
*C3*	19	Hom (106/106)	rs406514	0.761	intron	Benign	complement component 3 deficiency	Age-related macular degeneration, C3 deficiency

*ACMG: Association for Molecular Pathology, dbSNP: Single Nucleotide Polymorphism Database, Het: heterozygous, Hom: homozygous, OMIM: Online Mendelian Inheritance in Man, SNV: single nucleotide variant.*

**Table 3:** Primer design according to the provided accession number and locations.

Genes with Accession Number	Variant	Amino Acid Change	Primers
*BRCA2* (NM_000059)	c.7397T > C	p.V2466A	F: TTTCACAGAGTTGAACAGTGTGT R: AGGGCTTTAAAATTACCACCACC
*CFTR* (NM_000492)	c.1251C > A	p. N417K	F: CATGGGCCATGTGCTTTTCA R: CTTGCCTGCTCCAGTGGATC
*NCF1* (NM_000265)	c.295G > A	p.G99S	F:CTGCCCTCCCAGCCCCTCTCGGGCTT R: CGCACCTTGAAGAAGTCGAGGAG INT: CGCACCTTGAAGAAGTCGAGGAG
*PTPN22* (NM_015967)	c.1858T > C	p.W620R	F: ACTGATAATGTTGCTTCAACGGA R: AGGCTCACACATCAGCTTCC
*C3* (NM_000064)	c.2246-8C > T		F: CTCCAACTCCTGGCCTCAAG R: CGGTGGCTCTTTCAAGTCCT

*F: forward, R: reverse*.

## Data Availability

All data generated or analyzed during this study are included in this published article and its Supplementary Information files.

## Supplementary Materials

The following supporting information can be downloaded at: https://www.mdpi.com/article/10.3390/ijms26010425/s1.
