# Supplementary material for: Whole-Exome Sequencing: Discovering Genetic Causes of Granulomatous Mastitis
Source: Int J Mol Sci. 2025 Jan 6;26(1):425. doi: 10.3390/ijms26010425 (PMC11721990; doi:10.3390/ijms26010425)
Supplement: Supplementary file 1 [file ijms-26-00425-s001.zip › ijms-3360298-supplementary.pdf]

Supplementary Material

Supplementary Figure

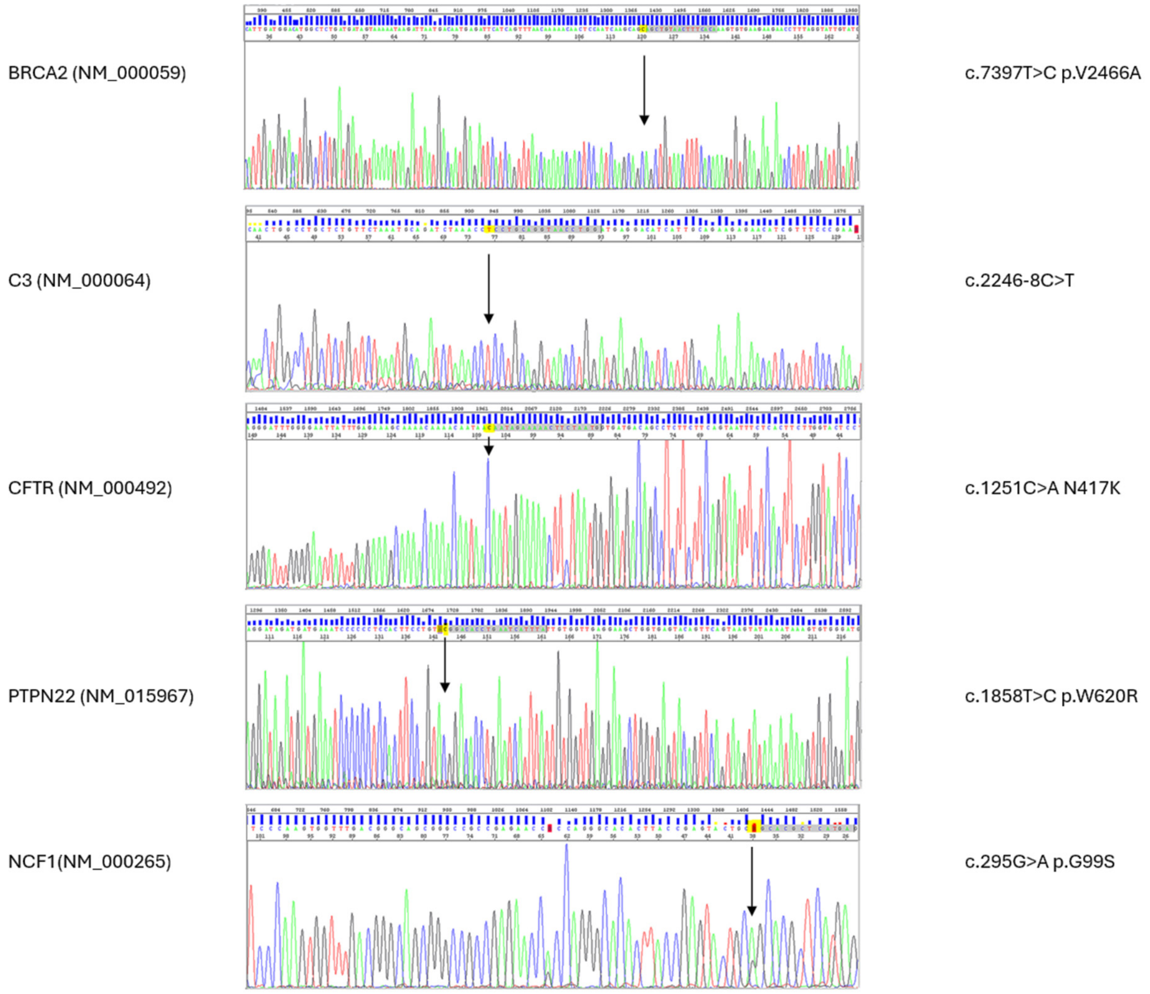

Supplementary Figure S1. This figure illustrates the DNA sequencing data obtained from NGS study highlighting variants in various genes.

The variants and their locations in the sequencing traces are indicated by black arrows:

1. ***BRCA2*** (NM_000059): Variant: c.7397T>C p.V2466A The sequencing trace shows a nucleotide change from T to C at position 7397, resulting in an amino acid substitution from Valine to Alanine at position 2466 in the *BRCA2* gene.
2. ***C3*** (NM_000064): Variant: c.2246-8C>T The sequencing trace indicates a nucleotide change from C to T at position 2246-8 in the *C3* gene.
3. ***CFTR*** (NM_000492): Variant: c.1251C>A N417K This trace shows a nucleotide change from C to A at position 1251, leading to an amino acid change from Asparagine to Lysine at position 417 in the *CFTR* gene.
4. ***PTPN22*** (NM_015967): Variant: c.1858T>C p.W620R The sequencing trace highlights a nucleotide change from T to C at position 1858, resulting in an amino acid substitution from Tryptophan to Arginine at position 620 in the *PTPN22* gene.
5. ***NCF1*** (NM_000265): Variant: c.295G>A p.G99S This trace shows a nucleotide change from G to A at position 295, leading to an amino acid substitution from Glycine to Serine at position 99 in the *NCF1* gene.
